# Supplementary material for: Mucosal host-microbe interactions associate with clinical phenotypes in inflammatory bowel disease
Source: Nat Commun. 2024 Feb 17;15:1470. doi: 10.1038/s41467-024-45855-2 (PMC10874382; doi:10.1038/s41467-024-45855-2)
Supplement: Supplementary file 1 — Supplementary Information [file 41467_2024_45855_MOESM1_ESM.pdf]

Supplemental Information

Supplemental Materials and Methods
Supplemental Results
Supplementary Fig. S1.
Supplementary Fig. S2.
Supplementary Fig. S3.
Supplementary Fig. S4.
Supplementary Fig. S5.
Supplementary Fig. S6.
Supplementary Fig. S7.
Supplementary Fig. S8.
Supplemental References

Supplemental Materials and Methods

Clinical data

Detailed phenotypic data were collected for all patients, including age, sex, BMI (body weight divided by squared height), smoking status, Montreal disease classification, medication usage, history of surgery, clinical disease activity and histological disease activity, and all were assessed at time of sampling. Medical treatment was noted irrespective of prescribed dosages, intervals or phase of treatment. Montreal disease classification was recorded from the closest visit to the outpatient clinic at time of sampling. Clinical disease activity was established using the Harvey-Bradshaw Index (HBI) for patients with CD and the Simple Clinical Colitis Activity Index (SCCAI) for patients with UC.

RNA isolation and RNA-seq data processing

RNA isolation was performed using the AllPrep DNA/RNA mini kit (Qiagen, reference number: 80204) according to manufacturer's instructions. Homogenization of intestinal biopsies was performed in RLT lysis buffer including β-mercaptoethanol using the Qiagen Tissue Lyser with stainless steel beads (diameter 5 mm, reference number: 69989). For the first sample batch, sample preparation was executed using the BioScientific NEXTflex™ Rapid Directional RNA-Seq Kit (Perkin-Elmer). Paired-end sequencing of RNA was performed using the Illumina NextSeq500 sequencer (Illumina). For the second sample batch, sample preparation was performed for construction of the Eukaryotic Transcriptome Library (Novogene). Paired-end sequencing of RNA was performed using the Illumina HiSeq PE250 platform. Sequencing was performed in two different batches, which necessitated pseudo-randomization (covering type of IBD diagnosis, biopsy location and disease activity) across plates to mitigate potential batch effects. The batch effects have been taken into account in all the analysis. On average, approximately 25 million reads were generated per sample.

Raw read quality was checked using FastQC with default parameters (ref v.0.11.7). Adaptors identified by FastQC were clipped using Cutadapt (ref v1.1) with default settings. Sickle (ref v1.200) was used to trim low-quality ends from the reads (length <25 nucleotides, quality <20). Reads were aligned to the human genome (human_glk_v37) using HISAT (ref v0.1.6) (with maximum allowance of two mismatches), and read sorting was performed using SAMtools (ref

v0.1.19). SAMtools flagstat and Picard tools (ref v2.9.0) were used to obtain mapping statistics. Six samples with low percentage read alignment (< 90%) were removed. Gene expression was estimated using HTSeq (ref v0.9.1), based on Ensemble version 75 annotation, resulting in a RNA expression dataset featuring 15,934 genes. Expression data on gene level were normalized using a trimmed mean of *M* values, and *clr* transformation was applied, resulting in 826 mucosal RNA-seq samples.

16S rRNA gene sequencing

Microbial composition of intestinal biopsies was determined by Illumina MiSeq paired-end sequencing of the V3-V4 hypervariable region of the 16S rRNA gene (MiSeq Benchtop Sequencer, Illumina Inc., San Diego, USA). Amplification of bacterial DNA was performed by PCR using modified 341F and 806R primers with a six-nucleotide barcode on the 806R primer for multiplexing. Sequences of both primers can be found in **Table S1**. Both primers contain an Illumina MiSeq adapter sequence, which is necessary for flow cell–binding in the MiSeq machine. Read trimming and filtering was done using *Trimmomatic* (0.33) to obtain an average read quality of 25 and a minimum length of 50. Quality was further checked using R package *DADA2* (v1.03) with the following parameters: minLen = 160, maxN=0, maxEE=c(2,2), truncQ=2 and rm.phix=TRUE. After error correction and chimera removal, the amplicon sequence variants were assigned to the *silva* database (v.132). Samples were rarefied at 2,000 mapped reads and those higher than this threshold were used for downstream analysis, resulting in 755 mucosal 16S samples. We further removed bacteria with low-abundance rate at 1% and low-present rate 10%, and kept 131 taxa for analysis. After accounting for overlap between mucosal RNA-seq and mucosal 16S data, 697 intestinal biopsies from 335 different patients and 16 non-IBD controls were available for host–microbiota interaction analyses. There was no significant effect of sample storage time on neither mucosal 16S nor bulk RNA-sequencing quality (**Fig. S8**).

Polymerase chain reaction (PCR), DNA clean-up, and MiSeq library preparation for mucosal 16 microbiota characterization

The PCR procedure consisted of the following conditions: an initial cycle of 94°C for 3 min followed by 32 cycles of 94°C for 45 sec, 50°C for 60 sec and 72°C for 90 sec, with a final extension of 72°C for 10 min. Agarose gel electrophoresis confirmed the presence of the PCR product (band

at ~465 bp) in successfully amplified samples. Subsequently, DNA samples were thoroughly cleaned by mixing the remainder of the PCR product with 25 µL Agencourt AMPure XP beads (Beckman Coulter, Brea, California, USA) followed by an incubation of 5 min at room temperature. Beads were separated from the mixture by placing the samples within a magnetic bead separator for 2 min. After discarding the cleared solution, beads were washed twice by resuspending them in 200 µL fresh 80% ethanol, followed by an incubation of 30 sec in the magnetic bead separator, and again discarding the cleared solution. The pellet was dried for 15 min and resuspended in 52.5 µL 10 mM Tris HCl buffer (pH 8.5). Fifty (50) µL of this solution was subsequently brought into a new tube. DNA concentrations were measured using a Qubit® 2.0 fluorometer (Thermo-Fisher Scientific, Waltham, Massachusetts, USA). To ensure similar library representations across samples, 2 nM dilutions of each sample were prepared accordingly. A library was created by pooling 5 µl of each diluted sample. Subsequently, 10 µL of the sample pool and 10 µL 0.2 M NaOH were mixed and incubated for 5 min to allow denaturation of the sample DNA. 980 µL of the HT1 buffer of the MiSeq 2x300 cartridge was then added to this mixture. Next, a denatured diluted PhiX solution was created by combining 2 µL 10 nM PhiX library with 3 µL 10 mM Tris HCl buffer (pH 8.5) with 0.1% Tween-20. 5 µL 0.2 M NaOH was added to this mixture and incubated for 5 min at room temperature. This 10 µL mixture was eventually mixed with 990 µL HT1 buffer. From the diluted sample pool, 150 µL was combined with 50 µL of the diluted PhiX solution, which was further diluted by the addition of 800 µL HT1 buffer. Finally, 600 µL of the prepared library solution was loaded into the sample loading reservoir of the 2x300 MiSeq cartridge for 16S rRNA amplicons sequencing (MiSeq Benchtop Sequencer, Illumina, San Diego, California, USA). Samples with low DNA concentrations after clean-up (quality score < 0.9) were discarded by PANDAseq to increase quality of sequence read-outs.

Mucosal gene expression analysis and microbial characterization – statistical analysis

To assess the effect of mucosal inflammation on gene expression, we re-coded the inflammation status in an ordinal fashion as 0, 1 or 2 to represent biopsies from non-IBD controls, biopsies from non-inflamed tissue of patients with IBD and biopsies from inflamed areas of patients with IBD, respectively. Intestinal inflammatory status was thus treated as a continuous variable to account for presence of residual inflammation in biopsies marked as being taken from non-inflamed areas in the intestines. A correction for all multiple tests from three groups was applied using an BH-adjusted *P* threshold of 0.05.

1) Inflammation-associated genes were identified in three comparisons: (1) CD colonic inflamed tissue vs. CD colonic non-inflamed tissue vs. non-IBD colonic tissue, (2) CD ileocecal inflamed tissue vs. CD ileocecal non-inflamed tissue vs. non-IBD ileocecal tissue and (3) UC colonic inflamed tissue vs. UC colonic non-inflamed tissue vs. non-IBD colonic tissue:

Gene ~ intercept + inflammation + age + sex + BMI + medication + batch + (1|ID)

2) Clinical phenotype–associated genes were identified using the following model:

Gene ~ intercept + Montreal/anti-TNF therapy + age + sex + BMI + inflammation + tissue location + medication + batch + (1|ID)

Microbial characterization

Associations between microbial features and biopsy inflammatory status, IBD diagnosis, disease location (biopsy origin) and clinical phenotypes were performed using general linear models (see below). Per sample, the mucosal dysbiosis score was defined as the median Aitchison distance from that sample to a reference sample set of non-IBD controls. Dysbiotic status was defined as being at the 90th percentile of this score [8].

1) Associations between microbial taxa and biopsy inflammation/location:

Taxa ~ intercept + inflammation + location + age + sex + BMI + medication + batch + surgical resection + (1|ID)

2) Associations between microbial taxa and clinical phenotypes:

Taxa ~ intercept + Montreal/anti-TNF therapy + inflammation + location + age + sex + BMI + medication + batch + surgical resection + (1|ID)

Supplemental Results

Table 1. Demographic and clinical characteristics of the study population compared between the inflamed and non-inflamed dataset.

Variable	Total	IBD		Non-IBD	*P*-value
		Inflamed biopsies	Non-inflamed biopsies	Controls	
**Biopsy level**	*n* = 697	*n* = 211	*n* = 434	*n* = 52	
Biopsy inflammation, *n* (%)					
Inflamed	211 (30.3)	211 (100)	-	-	
Non-inflamed	434 (62.3)	-	434 (100)	-	
Biopsy location, *n* (%)					
Ileum	245 (35.2)	66 (30.8)	171 (39.4)	9 (17.3)	
Colon	452 (64.8)	146 (69.2)	263 (60.6)	43 (82.7)	
Diagnosis or control, *n* (%)					0.81
CD	356 (51.1)	115 (54.5)	241 (55.5)	-	
UC	289 (41.5)	96 (45.5)	193 (44.5)	-	
Controls	52 (7.5)	-	-	52 (100)	
Age at biopsy (years)	42.9 ± 15.3	42.9 ± 16.1	42.7 ± 15.4	44.8 ± 10.7	0.65
Sex, *n* (%)					<0.01
Male	317 (45.5)	90 (42.7)	188 (43.3)	39 (75.0)	
Female	380 (54.5)	121 (57.3)	246 (56.7)	13 (25.0)	
BMI (kg/m^2)	25.6 ± 4.5	25.7 ± 4.6	25.7 ± 4.6	24.6 ± 2.5	0.28
Current smoking, *n* (%)					<0.01
Yes	147 (21.1)	36 (17.1)	91 (21.0)	20 (38.5)	
No	550 (78.9)	175 (82.9)	343 (79.0)	32 (61.5)	

Variable	IBD	CD	UC	Non-IBD Controls	*P*-value
**Individual (patient) level**	*n* = 335	*n* = 181	*n* = 154	*n* = 16	
Age (years)	43.1 ± 15.4	41.4 ± 15.2	45.2 ± 15.4	44.4 ± 11.5	
Sex, n (%)	335 (100)	181 (100)	154 (100)	16 (100)	<0.001
Male	147 (43.9)	63 (34.8)	84 (54.54)	12 (75.0)	
Female	188 (56.1)	118 (65.2)	70 (45.5)	4 (25.0)	
BMI (kg/m^2)	25.9 ± 4.5	25.6 ± 4.7	26.3 ± 4.3	24.6 ± 2.9	
Current smoking, *n* (%)	335 (100)	181 (100)	154 (100	16 (100)	0.004
Yes	69 (20.6)	48 (26.5)	21 (13.6)	6 (37.5)	
No	266 (79.4)	133 (73.5)	133 (86.4)	10 (62.5)	
Montreal Age (A), *n* (%)	330 (98.5)	180 (	150 (	-	
A1 (≤16 years)	36 (10.9)	27 (15.0)	9 (6.0)	-	
A2 (17–40 years)	211 (63.9)	119 (66.1)	92 (61.3)	-	
A3 (>40 years)	83 (25.2)	34 (18.9)	49 (32.7)	-	
Montreal Location (L), *n* (%)		181 (100)	-	-	
L1 (ileal disease)		37 (20.4)	-	-	
L2 (colonic disease)		32 (17.7)	-	-	
L3 (ileocolonic disease)		87 (48.0)	-	-	
L1 + L4		6 (3.3)	-	-	
L2 + L4		3 (1.7)	-	-	
L3 + L4		16 (8.8)	-	-	
Montreal Behavior (B), *n* (%)		181 (100)	-	-	
B1 (non-stricturing, non-penetrating)		75 (41.4)	-	-	
B2 (stricturing)		31 (17.1)	--	-	

B3 (penetrating)		17 (93.9)	-	-	
B1 + P (perianal disease)		25 (13.8)	-	-	
B2 + P (perianal disease)		24 (13.3)	-	-	
B3 + P (perianal disease)		9 (5.0)	-	-	
Montreal Extension (E), *n* (%)		-	135 (87.7)	-	
E1 (proctitis)		-	10 (7.4)	-	
E2 (left-sided colitis)		-	45 (33.3)	-	
E3 (pancolitis)		-	80 (59.3)	-	
Montreal Severity (S), *n* (%)		-	112 (72.7)	-	
S0 (remission)		-	5 (4.5)	-	
S1 (mild)		-	14 (12.5)	-	
S2 (moderate)		-	63 (56.3)	-	
S3 (severe)		-	30 (26.8)	-	
**Medication use**					
Aminosalicylates, *n* (%)	140 (41.8)	18 (9.9)	122 (79.2)	-	<0.01
Thiopurines, *n* (%)	110 (32.8)	61 (33.7)	49 (31.8)	-	0.71
Steroids, *n* (%)	127 (37.9)	63 (34.8)	64 (41.6)	-	0.20
Methotrexate, *n* (%)	21 (6.3)	16 (8.8)	5 (3.2)	-	0.04
TNF-α-antagonists, *n* (%)[†]	59 (17.6)	48 (26.5)	11 (7.1)	-	<0.01
**Clinical disease activity**					
HBI		179 (98.9)	-	-	
Remission (<5)		124 (69.3)	-	-	
Active disease (≥5)		51 (28.5)	-	-	
SCCAI		-	137 (90.0)	-	
Remission (≤2)		-	81 (59.1)	-	

Active disease (>2)		-	56 (40.9)	-	
**Surgical history**					
Ileocecal resection, *n* (%)	72 (21.5)	70 (38.7)	2 (1.3)	-	<0.01
Colon resection (or partial), *n* (%)	72 (21.5)	44 (24.3)	28 (18.2)	-	0.17
Small intestinal (partial) resection, *n* (%)	37 (11.0)	36 (19.9)	1 (0.6)	-	<0.01

Data are presented as proportions *n* with corresponding percentages (%), mean ± standard deviation (SD) or as median [interquartile range, IQR] in case of continuous variables. *P* values of comparing categorical variables between groups are from two-sided χ2 test; *P* values of comparing continuous variable between two groups are from two-sided Wilcoxon's test. *P*-values ≤ 0.05 were considered statistically significant. †Use of TNF-α-antagonists included use of infliximab, adalimumab, golimumab and certolizumab pegol. Abbreviations: BMI, body-mass index; CD, Crohn's disease; HBI, Harvey-Bradshaw Index; IBD, inflammatory bowel disease; TNF-α, tumor necrosis factor alpha; SCCAI, Simple Clinical Colitis Activity Index; UC, ulcerative colitis.

Box 1. Individual mucosal gene–bacteria associations and their potential biological implications in IBD.

Mucosal bifidobacteria positively associate with aryl hydrocarbon receptor (AHR) and ABC-transporter (ABCC1) expression levels

The positive association between *AHR* expression and bifidobacteria, although in the absence of an association between *CYP1A1* expression an bifidobacteria, could be explained by the fact that *Bifidobacterium* spp. can produce aromatic lactic acids such as indole-3-lactic acid (out of aromatic amino acids like tryptophan) via aromatic lactate dehydrogenase, which in turn activates the host aryl hydrocarbon receptor [1,2]. Activation of the aryl hydrocarbon receptor, a crucial regulator of intestinal homeostasis and immune responses, leads to a reduction of inflammation in intestinal epithelial cells [3] and confers immunoprotective effects [4].

Another intriguing observation is the positive association between bifidobacteria and host expression of the *ABCC1* gene. *ABCC1* is a member of the ATP-binding cassette transporters (ABC transporters, and also known as multidrug resistance-associated protein 1, MRP1) that has

multiple physiological functions, but it may also confer pathophysiological sequelae, especially in the context of cancer [5]. Under physiological circumstances, it detoxifies endogenously generated toxic substances (as well as xenobiotics), protects against oxidative stress, transports leukotrienes and lipids and may facilitate the cellular export and body distribution of vitamin B_{12} [6]. Interestingly, several *Bifidobacterium* species (e.g. *B. animalis*, *B. longum* and *B. infantis*) can synthesize vitamin B_{12}, which is subsequently absorbed in the large intestine via unknown mechanisms [7-9].

Mucosal bifidobacteria associate with FOSL1, a subunit of the AP-1 transcription factor

Associations between mucosal *Bifidobacterium* bacteria and expression of *FOSL1* genes were amongst the top significant individual gene–bacteria interactions. Fos-related antigen 1 (FRA1), encoded by *FOSL1*, is a subunit of the activator protein 1 (AP-1) transcription factor. In the intestine, the AP-1 transcription factor is commonly activated in response to inflammatory stimuli and has been implicated in IBD [10]. More specifically, an interaction may exist between AP-1 activity and the glucocorticoid receptor, which may be part of the anti-inflammatory effects of steroid treatment [11]. In steroid-resistant patients with CD, AP-1 activation was primarily observed in the nuclei of intestinal epithelial cells, whereas this activation was restricted to lamina propria macrophages in steroid-sensitive patients [10]. This suggests a differing cellular activation pattern of AP-1 activation in steroid-resistant patients where the expression of this transcription factor may interfere with the activity of the glucocorticoid response. In an experimental study in which pregnant mice were supplemented with butyrate, FOS genes, including *Fosl1*, were observed to be downregulated in the colon and associated with protection against experimentally-induced colitis [12]. Although there are currently no reports of potential immune-modulating effects for Fosl1, it has 85% homology with Fosl2, another AP-1 transcription factor. A recent study demonstrated that Fosl2 is important in T-reg development and control of autoimmunity. Interestingly, several GWASs have reported associations of a SNP located in the promoter region of *FOSL2* with IBD [13-15], and the presence of this SNP was also shown to correlate with *FOSL2* expression in blood cells of patients with IBD [16]. In the context of T-regs, *FOSL2* also appears to be important as it is a determinant of a highly suppressive subpopulation of T-regs in humans that are particularly enriched in the lamina propria of patients with CD, supporting wound healing in the intestinal mucosa [17]. Although speculative, bifidobacteria, as well as their metabolites such as butyrate, may potentially confer immune-modulating properties via interaction with *FOSL1* expression.

Mucosal bifidobacteria positively associate with Krüppel-like factor 2 (KLF2) expression

Krüppel-like factor 2 (encoded by *KLF2*) is a negative regulator of intestinal inflammation, and its expression is found to be reduced in patients with IBD [18]. *KLF2* also negatively regulates differentiation of adipocytes and strongly inhibits PPAR-γ expression, which prevents differentiation of preadipocytes into adipocytes and thereby prevents adipogenesis [19]. *KLF2* also plays an important role in endothelial physiology, where it may act as a molecular switch by regulating endothelial cell function in inflammatory disease states [20]. Interestingly, *KLF2* modifies the trafficking of T-regs, as increased *KLF2* expression in T-regs promotes the induction of peripheral immunological tolerance, whereas, in the absence of its expression, T-regs are unable to effectively migrate to secondary lymphoid tissues [21]. Indeed,, it was demonstrated in mouse experiments that mice developed IBD in the presence of *KLF2*-deficient T-regs, which were unable to prevent colitis by disrupted co-trafficking of effector and regulatory T cells. In light of these considerations, mucosal bifidobacteria may confer beneficial immune-modulating properties by upregulating *KLF2* expression, thereby stimulating T-reg migration and contributing to immunological self-tolerance in the context of IBD.

Mucosal Anaerostipes bacteria positively associate with host SMAD4 expression

Anaerostipes, which belong to the *Lachnospiraceae* family, are anaerobic bacteria that are well-known butyrate-producers. Butyrate serves as the primary energy source for colonic epithelial cells and is characterized by anti-inflammatory and anti-carcinogenic properties. *SMAD4* is an important intracellular effector of the TGF-β superfamily of proteins. These proteins have important functions in alleviating intestinal inflammation and maintenance of gut mucosal homeostasis. Haploinsufficiency of *SMAD4* in mice and humans has been associated with an increased susceptibility to colonic inflammation [22]. In patients with CD, reduced epithelial protein levels of SMAD4 were observed that was associated with disease activity, indicating defective mucosal TGF-β signaling during active intestinal inflammation. In an experimental animal study, mice with an epithelial deletion of *Smad4* presented with macroscopic invasive adenocarcinoma of the distal colon and rectum 3 months after DSS-induced colitis [23]. Indeed, *SMAD4* mutations in humans are linked to juvenile polyposis syndrome and associated with poor disease outcome in several types of cancer [24-27]. Using RNA-seq analysis, a strong inflammatory expression profile was observed after *SMAD4* deletion, with expression of various inflammatory cytokines and chemokines, including CCL20. In addition, it was demonstrated that CCL20 could be repressed by *SMAD4* in colonic epithelial cells, proving that TGF-β signaling could block the induction of CCL20 expression to protect against the development of colitis-associated cancer.

In an experimental study involving human hepatic stellate cells, butyrate was demonstrated to be protective against diet-induced nonalcoholic steatohepatitis and liver fibrosis via suppression of TGF-β signaling pathways in which SMAD proteins are involved. Although butyrate mainly showed antifibrotic effects via reduction of non-canonical TGF-β signaling cascades, there was also a significant increase in the expression of SMAD4 with the addition of butyrate on top of TGF-β treatment [28]. We found *Anaerostipes* bacteria to also be strongly associated with expression of *ZNF644*, a zinc finger protein that is positively regulated by intracellular zinc concentrations. Depletion of intracellular zinc levels, or even zinc deficiency, may have destabilizing effects on SMAD proteins and thereby impair the TGF-β signaling pathway [29].

Mucosal Verrucomicrobia bacteria inversely associate with expression of the IBD susceptibility gene YDJC

We observed significant inverse associations between Verrucomicrobia bacteria, of which *Akkermansia muciniphila* is a well-known member, and the expression of the *YDJC* gene, which encodes for the YdjC chitooligosaccharide deacetylase homolog (YdjC) protein. This gene has been identified as a shared susceptibility gene for CD, UC and psoriasis [13,30,31]. *YDJC* was originally identified as a celiac disease–associated susceptibility locus, but some SNPs were also associated with CD as well as with pediatric-onset CD [32]. YdjC catalyzes the deacetylation of acetylated carbohydrates, an important reaction in the degradation of oligosaccharides [33]. *YDJC* expression has been associated with tumor progression in studies of lung cancer [34,35]. The observed inverse association between *Akkermansia* and *YDJC* expression may suggest a potential protective role of *Akkermansia*, as decreased *YDJC* expression may mitigate its pro-carcinogenic effects. Despite the association between *YDJC* and the susceptibility to IBD on a genetic level, its precise functional role remains largely unknown [32].

Mucosal Alistipes bacteria positively associate with MUC4 expression

The bacterial genus *Alistipes*, belonging to family *Rikenellaceae* and phylum Bacteroidetes, is a recently discovered bacterial species, of which many have been isolated from the human gut microbiome. The role of *Alistipes* in health and disease is still unclear. Some evidence indicates that it may confer protective effects to the host, but other studies report pathogenic effects, e.g. in colorectal cancer development. A key factor believed to determine the relative abundance of *Alistipes* is the dysbiotic state of the gut microbiome [36]. In IBD, there is also conflicting data about the pathogenicity of *Alistipes* species. *Alistipes finegoldii* has been demonstrated to exert anti-inflammatory effects in experimental models of colitis [37]. Likewise, another study found an increased abundance of *Alistipes* in *NOD2*-knockout mice that had less severe (TNBS-induced)

colitis compared to wild-type mice [38]. It has also been reported that *Alistipes* abundance could increase after taking probiotic supplements, which in turn may protect against hepatocellular cancer growth in an experimental setting [39]. However, metagenomic studies have shown that *Alistipes* abundances were increased in mouse models of spontaneous CD-like ileitis terminalis as compared to wild-type mice, suggesting that *Alistipes* species may also play a pathogenic role by eliciting segmental ileitis [40,41].

MUC4 encodes for mucin 4, a protein found in the glycocalyx present on the intestinal epithelium. Deletion or knockouts of *Muc4* have demonstrated protective effects in mouse models, as shown by lower levels of proinflammatory factors and resistance against DSS-induced colitis. It is still unclear how this protective mechanism of *MUC4* deletion works, but it has been hypothesized that it may trigger the concomitant upregulation of other mucin proteins (e.g. *MUC*1-3) as these genes have been observed to be highly expressed in *Muc4*-knockout mice with DSS-induced colitis [42,43]. Based on this, we speculate that the positive association between *Alistipes* abundance and *MUC4* expression may imply a potential pathogenic role of *Alistipes* in the context of IBD-associated dysbiosis. However, in our data, we did not observe a significant interaction via dysbiotic status between *Alistipes* abundance and *MUC4* expression.

Mucosal Oscillibacter bacteria positively associate with OSM expression

Oscillibacter-like bacteria, which include *Oscillibacter* and *Oscillospira*, are commonly detected in human gut microbial communities, although their exact physiological role is not fully understood. Previously, it was reported that *Oscillibacter* may be a potentially important bacterium in mediating high fat diet–induced intestinal dysfunction, which was supported by a negative association between *Oscillibacter* and intestinal barrier function parameters [44]. Similarly, the abundance of *Oscillibacter* has been reported as a key bacterial group associated with colitis development in DSS-induced colitis in mice and with prenatal stress in rodents [45,46]. However, a recent study linking gut microbiota profiles to sulfur metabolism in patients with CD demonstrated that *Oscillibacter* abundance was enriched in patients with inactive compared to active disease but diminished in patients with IBD compared to controls [47,48]. Thus, similar to *Bacteroides* and *Alistipes*, the exact functional role of *Oscillibacter* in the context of IBD remains elusive, but it will likely depend on gut microbial dysbiosis and the intestinal (inflammatory) environment. The *OSM* gene encodes for the oncostatin M protein, a well-known inflammatory mediator in IBD that drives intestinal inflammation, mainly *via* activation of JAK-STAT and PI3K-Akt pathways [49]. Besides induction of other inflammatory events, it primarily triggers the production of various cytokines, chemokines and adhesion molecules that contribute to intestinal inflammation [50]. In addition,

OSM is a marker for non-responsiveness to TNF-α-antagonists in patients with IBD [51]. Considering these findings, the positive association between *OSM* expression and *Oscillibacter* abundance we observe supports a potentially pathogenic role for this bacterial species in IBD.

Mucosal Blautia bacteria associate with host ST13 expression levels

Hsc70-interacting protein, encoded by the *ST13* gene, mediates the assembly of the human glucocorticoid receptor, which requires involvement of intracellular chaperone proteins such as heat shock proteins HSP70 and HSP90 [52]. Reduced expression of ST13 has been observed in patients with colorectal cancer, suggesting that ST13 may constitute a candidate tumor-suppressor gene [53,54]. The positive association we observe between mucosal *Blautia* abundance and *ST13* gene expression may therefore point to a protective anti-carcinogenic role for *Blautia* in the intestines.

Downsampling analysis to confirm the sample size effect

To assess the sample size effect on results, we have performed the following assessments:

1. *Downsampling non-inflamed tissue samples to match the number of inflamed samples*

First, we down-sampled the number of non-inflamed biopsies (n=434) 10 times to match the number inflamed biopsies (n=211), and then repeated the sparse-CCA analysis and individual gene-bacteria associations tests with the same models and the same *P* value correction method (BH). In general, the gene modules (adjusted $P<0.05$) from sparse-CCA showed very good overlap rates varying from 58-71%, albeit the bacterial modules showed a bit weaker replication with rates varying from 38-69%. Moreover, the number of significant down-sampled individual gene-bacteria results (adjusted $P<0.05$) were lower, which were 16, 13, 9, 7, 0, 2, 15, 26, 0 and 33, but with on average a 75.86% overlap. All analyses here indicate that albeit the larger sample size increases the chance to identify more significant signals, it does not dramatically influence the main gene-bacteria association patterns in non-inflamed biopsies.

2. *Downsampling samples of patients without fibrostenotic CD to match fibrostenotic CD*

Subsequently, we down-sampled the amount of samples from patients without fibrostenotic CD (n=244) to match the number of samples from patients with fibrostenotic CD (n=107) for 10 times and repeated the network- and comparative analysis using the same methods. Here,

we identified the same four distinct microbiota associated gene clusters between the two groups (represented by *Lachnoclostridium*, *Coprococcus*, *Erysipelotrichaceae* and *Flavonifractor*). These four clusters showed significance in 8 out of 10 times downsampling rounds, indicating the patterns were quite stable. The *Faecalibacterium*-associated gene cluster was significant in 5 out of 10 times downsampling rounds, presumably because of a sample-size effect.

3. *Downsampling samples without TNF-α-antagonists usage to match samples under the usage of TNF-α-antagonists*

We down-sampled the samples from patients without TNF-α-antagonists usage (n=583) to match the number of samples from patients with TNF-α-antagonists usage (n=113) for 10 times and repeated the network and comparison analysis using the same methods. In general, only the *Ruminococcaceae*-UCG_002 associated gene cluster was still present across downsampling tests, which showed significance in 7 out of 10 times downsampling rounds. The *Faecalibacterium* and *Ruminococcaceae*_UCG-005 associated gene clusters were largely influenced by sample size.

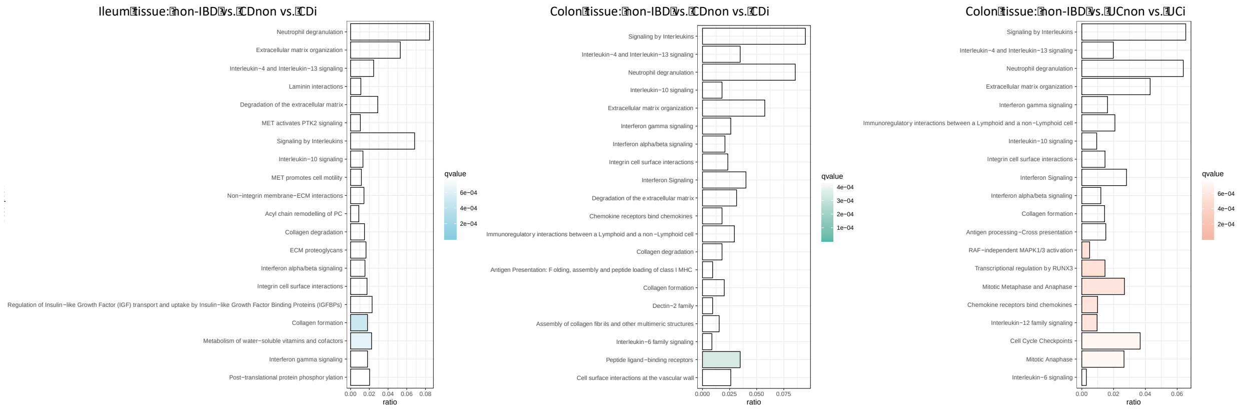

Supplementary Fig. S1. **Analysis of pathways associated with each comparative gene expression analysis.** The main pathways associated with inflamed ileal tissue in patients with CD (blue) include neutrophil degranulation, extracellular matrix (ECM) organization and IL-4/IL-13-signaling. Similar pathways were overexpressed in inflamed colonic tissue from patients with CD (green), but with a more prominent contribution from interleukin signaling pathways. Interleukin signaling pathways were also dominantly expressed in inflamed colonic tissue from patients with UC (orange), with other pathways expressed including neutrophil degranulation, ECM pathways, interferon gamma signaling and immunoregulatory interactions between lymphoid and non-lymphoid cells. Pathways were annotated using the Reactome pathway database. Abbreviations: CDi, inflamed tissue from patients with Crohn’s disease; CD-non, non-inflamed tissue from patients with Crohn’s disease; UCi, inflamed tissue from patients with ulcerative colitis; UC-non, non-inflamed tissue from patients with ulcerative colitis.

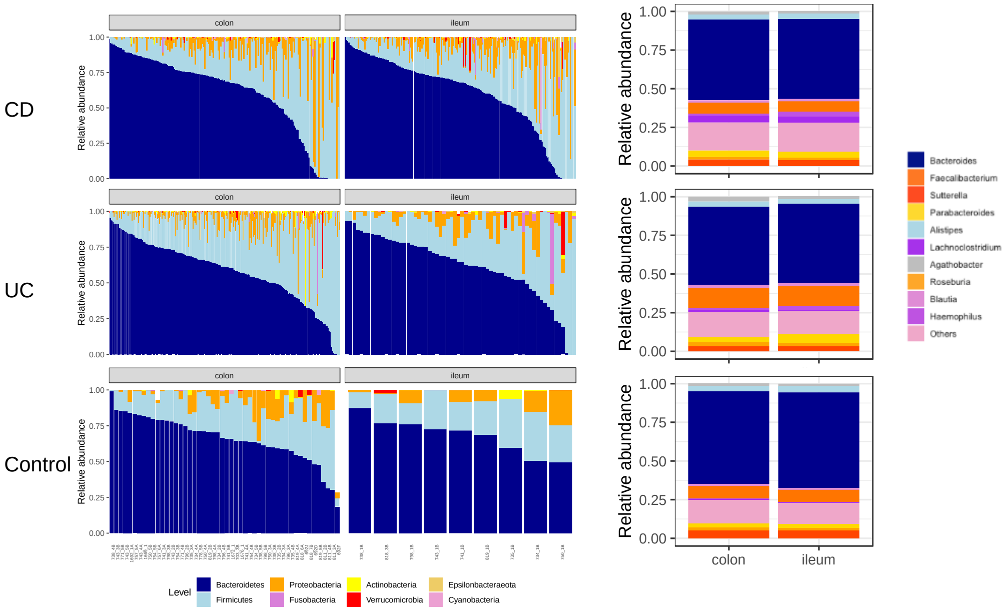

Supplementary Fig. S2. **Mucosal 16S rRNA gene sequencing characterization demonstrates distinct compositional differences in relative abundances** on (**A**) bacterial phylum level and (**B**) bacterial genus level. Abbreviations: CD, Crohn's disease; UC, ulcerative colitis.

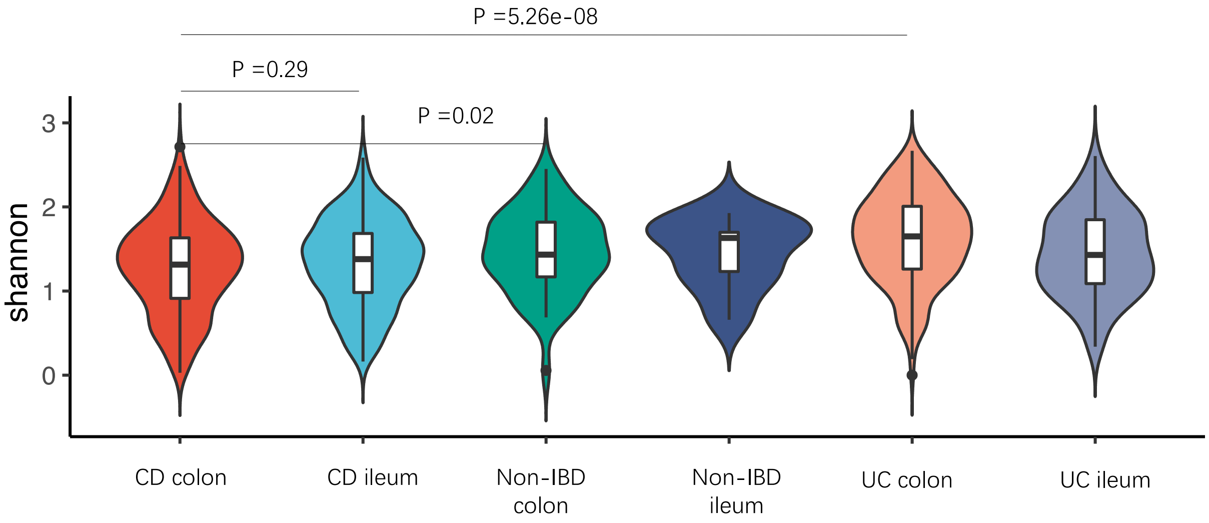

Supplementary Fig. S3. **Microbial alpha-diversity (Shannon index) is significantly lower in colonic biopsies from patients with CD compared to colonic biopsies derived from patients**

with UC or controls. This indicates that this difference is not solely attributable to ileal biopsies from patients with CD.

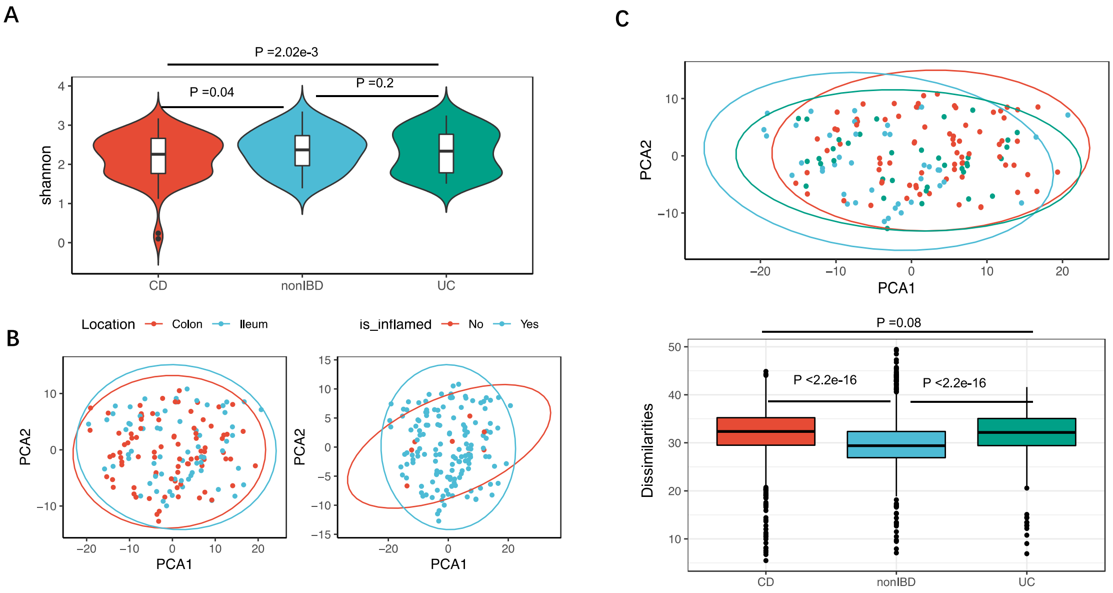

Supplementary Fig. S4. **Replication of overall mucosal microbiota characterization in patients with IBD and non-IBD controls.** Replication was performed in data derived from the HMP2 cohort study. **a**, Microbial alpha-diversity (Shannon index) was lowest in ptaients with CD (n=85) compared to patients with UC (n=46) and non-IBD controls (n=45). **b**, PCA plots based on Aitchison's distances and stratified by tissue location and inflammatory status (colors as in **a**). **c**, PCA plot showing microbial dissimilarity (Aitchison's distances) in CD, UC and non-IBD controls. **d**, Microbial dissimilarity is highest in samples from patients with CD, followed by patients with UC and non-IBD controls. CD, Crohn's disease; PCA, principal component analysis; UC, ulcerative colitis.

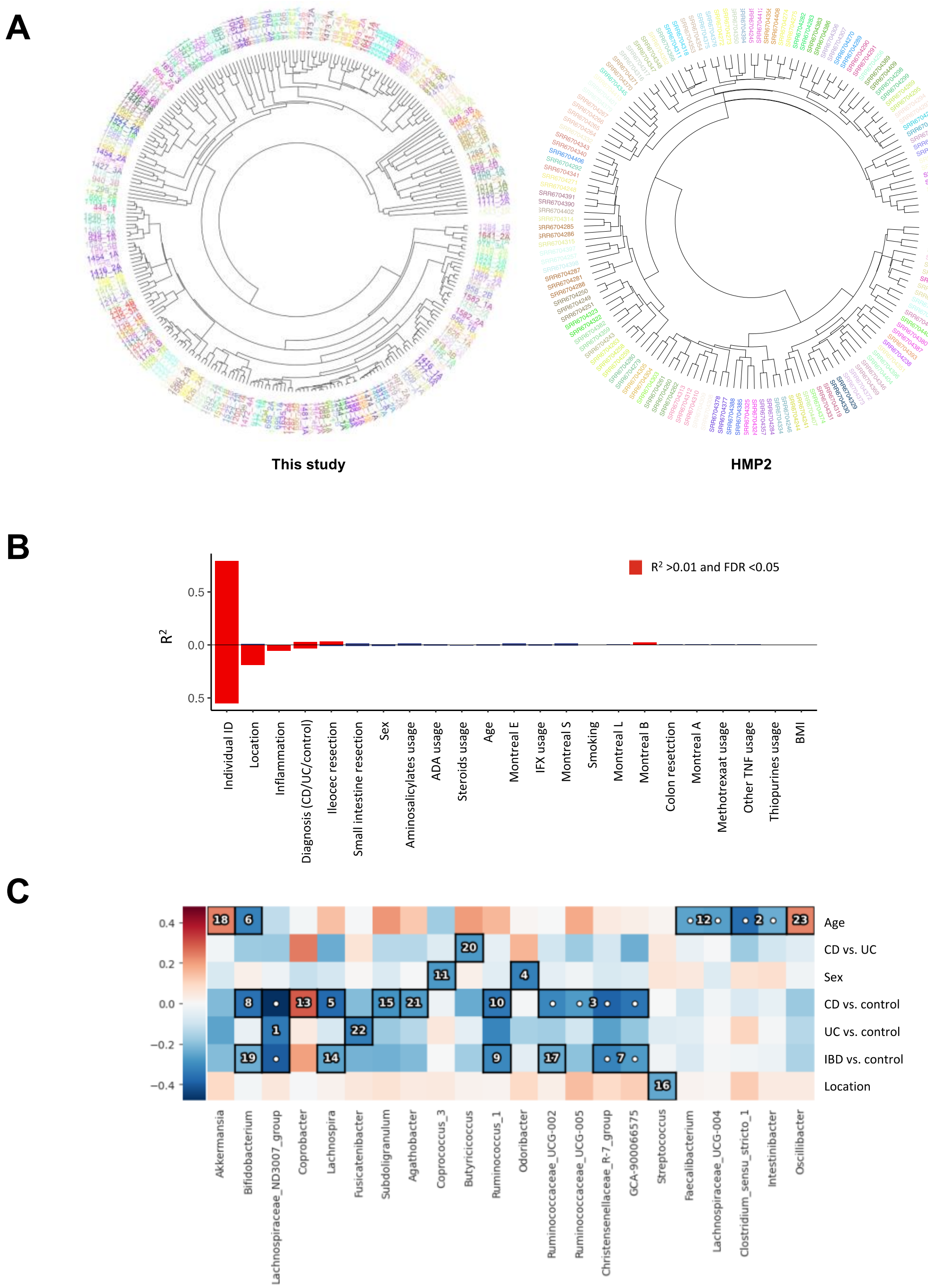

Supplementary Fig. S5. **Composition of the mucosal microbiota is highly personalized and influenced by disease parameters and clinical factors in patients with IBD and controls, using data derived from the HMP2 cohort [8].** (**A**) Hierarchical clustering analysis demonstrating that tissue samples from the same individual (paired samples) clearly cluster together (colors indicate unique individuals) in the HMP2 cohort. (**B**) Adonis analysis of

microbial community (genus level) and host gene expressions showed higher inter-individual variability in gut microbiota (R^2 =0.79, adjusted *P*<0.05). Neither the inflammation status nor tissue location explained >1% of the microbial variation while these two factors explained 6% and 19% variation in intestinal gene expressions, respectively. Red color indicates those factors significantly explain >1% variation (adjusted *P*<0.05). (**C**) Hierarchical analysis performed using an end-to-end statistical algorithm (HAllA) showing the main phenotypic factors that correlate with intestinal mucosal microbiota composition in the HMP2 cohort. Heatmap color palette indicates normalized mutual information. Numbers and dots in cells identify the significant pairs of features (phenotypic factors vs. bacterial taxa) in patients with IBD and controls. Abbreviations: BMI, body-mass index; CD, Crohn’s disease; UC, ulcerative colitis.

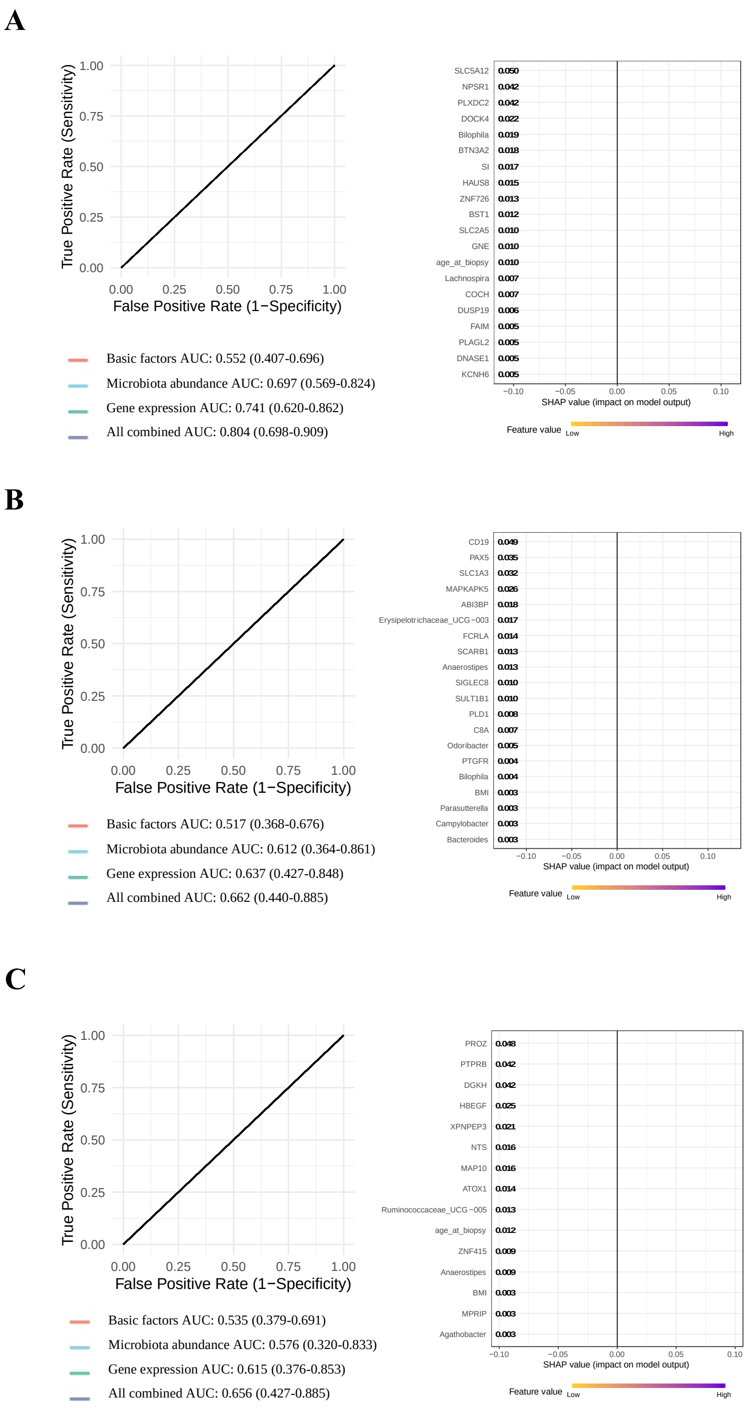

Supplementary Fig. S6. **Prediction of IBD subtypes.** Four different predictor combinations were used to predict CD vs. UC, Montreal B1 vs. B2 and Montreal E2 vs. E3 using eXtreme Gradient Boosting (xgboost) model. The clinical outcomes were selected based on the sample size. The predictors included age, sex, BMI, host gene expression and intestinal microbiota. SHapley Additive exPlanations (SHAP) values were calculated to quantify each feature

importance. A) right panel: models in discrimination between CD and UC, left panel: top 20 SHAP values, B) right panel: models in discrimination between Montreal B1 and B2, left panel: top 20 SHAP values, C) right panel: models in discrimination between Montreal E2 and E3, left panel: top 15 SHAP values. Model performance were only showed in test data set (20% samples).

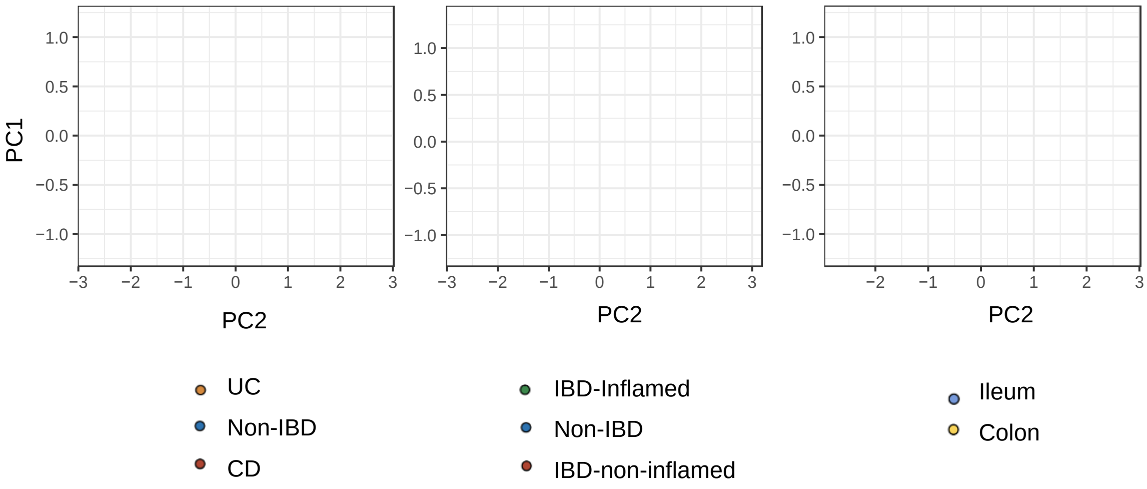

Supplementary Fig. S7. **Principal component analysis (PCA) plots demonstrating variation in cell type–enrichment labeled by diagnosis, biopsy inflammatory status and intestinal location**. Each dot represents one tissue sample. Left: Patients with IBD, both CD and UC, show significantly different intestinal cell type composition compared to controls. Middle: Tissue inflammatory status induces shifts in cell type composition, showing differences between non-inflamed IBD tissue vs. control tissue and inflamed IBD tissue vs. control tissue. Right: Tissue location (ileum vs. colon) also demonstrates distinct variation in cell type composition.

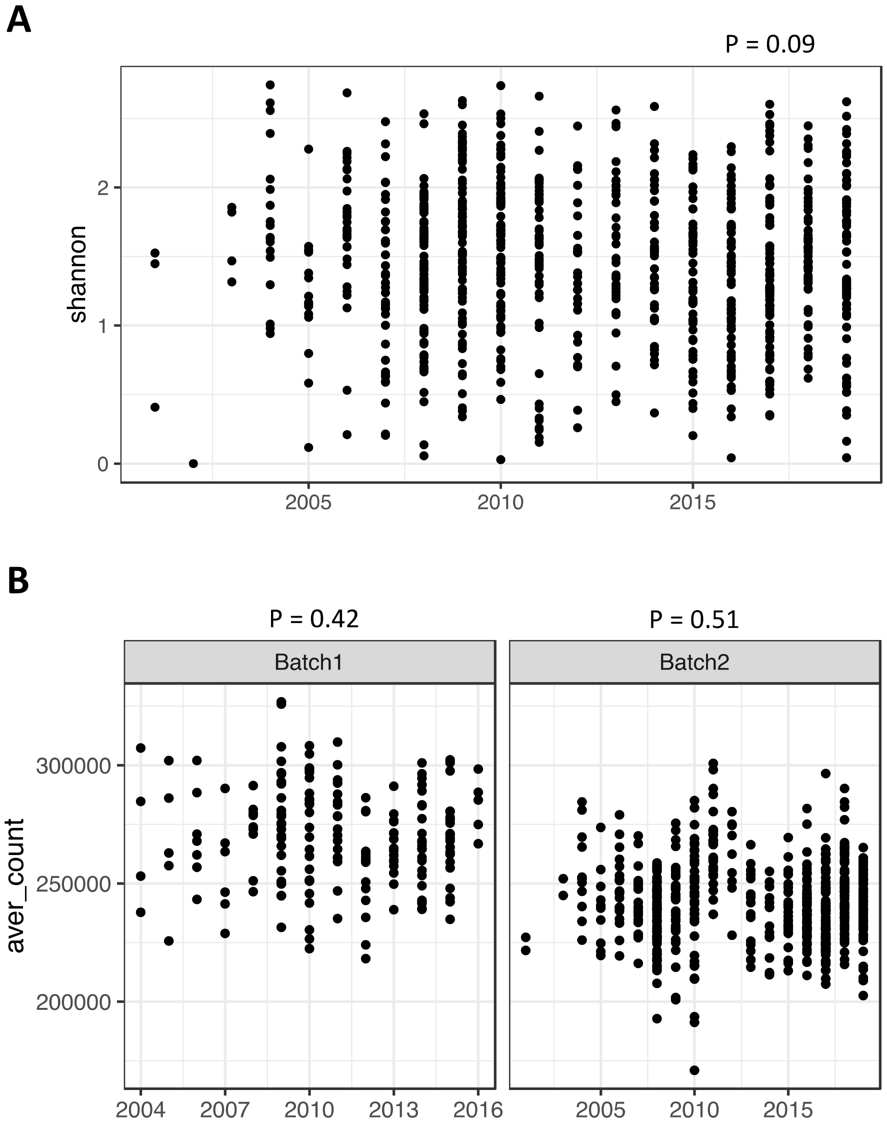

Supplementary Fig. S8. **Sample storage time and sequencing quality check.** (**A**) The correlation between sample collection year and Shannon diversity detected by mucosal 16S sequencing (Speaman correlation test, *P* =0.09). (**B**) The correlation between sample collection year and average gene count detected by mucosal bulk RNA sequencing in each batch (Spearman correlation test, *P* =0.42 in batch 1 and *P* =0.51 in batch 2).

Supplemental References to Box 1

1. Wong CB, Tanaka A, Kuhara T, et al. Potential effects of Indole-3-Lactic Acid, a Metabolite of Human Bifidobacteria, on NGF-induced Neurite Outgrowth in PC12 Cells. *Microorganisms* 2020;8(3):398. doi: 10.3390/microorganisms8030398.

2. Laursen MF, Sakanaka M, von Burg N, et al. Bifidobacterium species associated with breastfeeding produce aromatic lactic acids in the infant gut. *Nat Microbiol* 2021;6(11):1367-1382. doi: 10.1038/s41564-021-00970-4.

3. Ehrlich AM, Pacheco AR, Henrick BM, et al. Indole-3-lactic acid associated with Bifidobacterium-dominated microbiota significantly decreases inflammation in intestinal epithelial cells. *BMC Microbiol* 2020;20(1):357. doi: 10.1186/s12866-020-02023-y.

4. Henrick BM, Rodriguez L, Lakshmikanth T, et al. Bifidobacteria-mediated immune system imprinting early in life. *Cell* 2021;184(15):3884-3898.e11. doi: 10.1016/j.cell.2021.05.030.

5. Bakos E, Homolya L. Portrait of multifaceted transporter, the multidrug resistance-associated protein 1 (MRP1/ABCC1). *Pflugers Arch* 2007;453(5):621-41. doi: 10.1007/s00424-006-0160-8.

6. He SM, Li R, Kanwar JR, et al. Structural and functional properties of human multidrug resistance protein 1 (MRP1/ABCC1). *Curr Med Chem* 2011;18(3):439-81. doi: 10.2174/092986711794839197.

7. Yoshii K, Hosomi K, Sawane K, et al. Metabolism of Dietary and Microbial Vitamin B Family in the Regulation of Host Immunity. *Front Nutr* 2019;6:48. doi: 10.3389/fnut.2019.00048.

8. Beedholm-Ebsen R, van de Wetering K, Hardlei T, et al. Identification of multidrug resistance protein 1 (MRP1/ABCC1) as a molecular gate for cellular export of cobalamin. *Blood* 2010;115(8):1632-9. doi: 10.1182/blood-2009-07-232587.

9. Lee JH, O'Sullivan DJ. Genomic insights into bifidobacteria. *Microbiol Mol Biol Rev* 2010;74(3):378-416. doi: 10.1128/MMBR.00004-10.

10. Bantel H, Schmitz ML, Raible A, et al. Critical role of NF-kappaB and stress-activated protein kinases in steroid unresponsiveness. *FASEB J* 2002;16(13):1832-4. doi: 10.1096/fj.02-0223fje.

11. Karin M, Chang L. AP-1--glucocorticoid receptor crosstalk taken to a higher level. *J Endocrinol* 2001;169(3):447-51. doi: 10.1677/joe.0.1690447.

12. Barbian ME, Owens JA, Naudin CR, et al. Butyrate supplementation to pregnant mice elicits cytoprotection against colonic injury in the offspring. *Pediatr Res* 2021; doi: 10.1038/s41390-021-01767-1.

13. Jostins L, Ripke S, Weersma RK, et al. Host-microbe interactions have shaped the genetic architecture of inflammatory bowel disease. *Nature* 2012;491(7422):119-24. doi: 10.1038/nature11582.

14. Liu JZ, van Sommeren S, Huang H, et al. Association analyses identify 38 susceptibility loci for inflammatory bowel disease and highlight shared genetic risk across populations. *Nat Genet* 2015;47(9):979-986. doi: 10.1038/ng.3359.

15. Ye BD, McGovern DPB. Genetic variation in IBD: progress, clues to pathogenesis and possible clinical utility. *Exp Rev Clin Immunol* 2016;12(10):1091-107. doi: 10.1080/1744666X.2016.1184972.

16. Di Narzo AF, Peters LA, Argmann C, et al. Blood and Intestine eQTLs from an Anti-TNF-Resistant Crohn's Disease Cohort Inform IBD Genetic Association Loci. *Clin Transl Gastroenterol* 2016;7(6):e177. doi: 10.1038/ctg.2016.34.

17. Povoleri GAM, Nova-Lamperti E, Scottà C, et al. Human retinoic acid-regulated CD161+ regulatory T cells support wound repair in intestinal mucosa. *Nat Immunol* 2018;19(12):1403-1414. doi: 10.1038/s41590-018-0230-z.

18. Wang ZL, Wang YD, Wang K, et al. KFL2 participates in the development of ulcerative colitis through inhibiting inflammation via regulating cytokines. *Eur Rev Med Pharmacol Sci* 2018;2(15):4941-4948. doi: 10.26355/eurrev_201808_15633.

19. Banerjee SS, Feinberg MW, Watanabe M, et al. The Krüppel-like factor KLF2 inhibits peroxisome proliferator-activated receptor-gamma expression and adipogenesis. *J Biol Chem* 2003;278(4):2581-4. doi: 10.1074/jbc.M210859200.

20. Banerjee SS, Lin Z, Atkins GB, et al. KLF2 Is a novel transcriptional regulator of endothelial proinflammatory activation. *J Exp Med* 2004;199(10):1305-15. doi: 10.1084/jem.20031132.

21. Pabbisetty SK, Rabacal W, Volanakis EJ, et al. Peripheral tolerance can be modified by altering KLF2-regulated Treg migration. *Proc Natl Acad Sci U S A* 2016;113(32):E4662-70. doi: 10.1073/pnas.1605849113.

22. Szigeti R, Pangas SA, Nagy-Szakal D, et al. SMAD4 haploinsufficiency associates with augmented colonic inflammation in select humans and mice. *Ann Clin Lab Sci* 2012;42(4):401-8.

23. Means AL, Freeman TJ, Zhu J, et al. Epithelial Smad4 Deletion Up-Regulates Inflammation and Promotes Inflammation-Associated Cancer. *Cell Mol Gastroenterol Hepatol* 2018;6(3):257-276. doi: 10.1016/j.jcmgh.2018.05.006.

24. Wang Y, Xue Q, Zheng Q, et al. SMAD4 mutation correlates with poor prognosis in non-small cell lung cancer. *Lab Invest* 2021;101(4):463-476. doi: 10.1038/s41374-020-00517-x.

25. Mizuno T, Cloyd JM, Vicente D, et al. SMAD4 gene mutation predicts poor prognosis in patients undergoing resection for colorectal liver metastases. *Eur J Surg Oncol* 2018;44(5):684-692. doi: 10.1016/j.ejso.2018.02.247.

26. Miyaki M, Iijima T, Konishi M, et al. Higher frequency of Smad4 gene mutation in human colorectal cancer with distant metastasis. *Oncogene* 1999;18(20):3098-103. doi: 10.1038/sj.onc.1202642.

27. Lin LH, Chang KW, Cheng HW, et al. SMAD4 Somatic Mutations in Head and Neck Carcinoma Are Associated With Tumor Progression. *Front Oncol* 2019;9:1379. doi: 10.3389/fonc.2019.01379.

28. Gart E, van Duyvenvoorde W, Toet K, et al. Butyrate Protects against Diet-Induced NASH and Liver Fibrosis and Suppresses Specific Non-Canonical TGF-β Signaling Pathways in Human Hepatic Stellate Cells. *Biomedicines* 2021;9(12):1954. doi: 10.3390/biomedicines9121954.

29. Dong S, Tian Q, Zhu T, et al. SLC39A5 dysfunction impairs extracellular matrix synthesis in high myopia pathogenesis. *J Cell Mol Med* 2021;25(17):8432-8441. doi: 10.1111/jcmm.16803.

30. Ellinghaus D, Ellinghaus E, Nair RP, et al. Combined analysis of genome-wide association studies for Crohn disease and psoriasis identifies seven shared susceptibility loci. *Am J Human Genet* 2012;90(4):636-47. doi: 10.1016/j.ajhg.2012.02.020.

31. Ye BD, Choi H, Hong M, et al. Identification of Ten Additional Susceptibility Loci for Ulcerative Colitis Through Immunochip Analysis in Koreans. *Inflamm Bowel Dis* 2016;22(1):13-9. doi: 10.1097/MIB.0000000000000584.

32. Parmar AS, Lappalainen M, Paavola-Sakki P, et al. Association of celiac disease genes with inflammatory bowel disease in Finnish and Swedish patients. *Genes Immun* 2012;13(6):474-80. doi: 10.1038/gene.2012.21.

33. Verma SC, Mahadevan S. The chbG gene of the chitobiose (chb) operon of Escherichia coli encodes a chitooligosaccharide deacetylase. *J Bacteriol* 2012;194(18):4959-71. doi: 10.1128/JB.00533-12.

34. Kim EJ, Park MK, Kang GJ, et al. YDJC Induces Epithelial-Mesenchymal Transition via Escaping from Interaction with CDC16 through Ubiquitination of PP2A. *J Oncol* 2019;2019:3542537. doi: 10.1155/2019/3542537.

35. Kim EJ, Park MK, Byun HJ, et al. YdjC chitooligosaccharide deacetylase homolog induces keratin reorganization in lung cancer cells: involvement of interactions between YDJC and CDC16. *Oncotarget* 2018;9(33):22915-22928. doi: 10.18632/oncotarget.25145.

36. Parker BJ, Wearsch PA, Veloo ACM, et al. The Genus Alistipes: Gut Bacteria With Emerging Implications to Inflammation, Cancer, and Mental Health. *Front Immunol* 2020;11:906. doi: 10.3389/fimmu.2020.00906.

37. Dziarski R, Park SY, Kashyap DR, et al. Pglyrp-Regulated Gut Microflora Prevotella falsenii, Parabacteroides distasonis and Bacteroides eggerthii Enhance and Alistipes finegoldii Attenuates Colitis in Mice. *PLoS One* 2016;11(1):e0146162. doi: 10.1371/journal.pone.0146162.

38. Butera A, Di Paola M, Pavarini L, et al. Nod2 Deficiency in mice is Associated with Microbiota Variation Favouring the Expansion of mucosal $CD4^+$ LAP^+ Regulatory Cells. *Sci Rep* 2018;8(1):14241. doi: 10.1038/s41598-018-32583-z.

39. Li J, Sung CY, Lee N, et al. Probiotics modulated gut microbiota suppresses hepatocellular carcinoma growth in mice. *Proc Natl Acad Sci U S A* 2016;113(9):E1306-15. doi: 10.1073/pnas.1518189113.

40. Rodriguez-Palacios A, Kodani T, Kaydo L, et al. Stereomicroscopic 3D-pattern profiling of murine and human intestinal inflammation reveals unique structural phenotypes. *Nat Commun* 2015;6:7577. doi: 10.1038/ncomms8577.

41. Rodriguez-Palacios A, Harding A, Menghini P, et al. The Artificial Sweetener Splenda Promotes Gut Proteobacteria, Dysbiosis, and Myeloperoxidase Reactivity in Crohn's Disease-Like Ileitis. *Inflamm Bowel Dis* 2018;24(5):1005-1020. doi: 10.1093/ibd/izy060.

42. Grondin JA, Kwon YH, Far PM, et al. Mucins in Intestinal Mucosal Defense and Inflammation: Learning From Clinical and Experimental Studies. *Front Immunol* 2020;11:2054. doi: 10.3389/fimmu.2020.02054.

43. Das S, Rachagani S, Sheinin Y, et al. Mice deficient in Muc4 are resistant to experimental colitis and colitis-associated colorectal cancer. *Oncogene* 2016;35(20):2645-54. doi: 10.1038/onc.2015.327.

44. Lam YY, Ha CWY, Campbell CR, et al. Increased gut permeability and microbiota change associate with mesenteric fat accumulation and metabolic dysfunction in diet-induced obese mice. *PLoS One* 2012;7(3):e34233. doi: 10.1371/journal.pone.0034233.

45. Peng Y, Yan Y, Wan P, et al. Gut microbiota modulation and anti-inflammatory properties of anthocyanins from the fruit of Lycium ruthenicum Murray in dextran sodium sulfate-induced colitis in mice. *Free Radic Biol Med* 2019;136:96-108. doi: 10.1016/j.freeradbiomed.2019.04.005.

46. Golubeva AV, Crampton S, Desbonnet L, et al. Prenatal stress-induced alterations in major physiological systems correlate with gut microbiota composition in adulthood. *Psychoneuroendocrinology* 2015;60:58-74. doi: 10.1016/j.psyneuen.2015.06.002.

47. Metwaly A, Dunkel A, Waldschmitt N, et al. Integrated microbiota and metabolite profiles link Crohn's disease to sulfur metabolism. *Nat Commun* 2020;11(1):4322. doi: 10.1038/s41467-020-17956-1.

48. Ryan FJ, Ahern AM, Fitzgerald RS, et al. Colonic microbiota is associated with inflammation and host epigenomic alterations in inflammatory bowel disease. *Nat Commun* 2020;11(1):1512. doi: 10.1038/s41467-020-15342-5.

49. Chollangi S, Mather T, Rodgers KK, et al. A unique loop structure in oncostatin M determines binding affinity toward oncostatin M receptor and leukemia inhibitory factor receptor. *J Biol Chem* 2012;287(39):32848-59. doi: 10.1074/jbc.M112.387324.

50. Verstockt S, Verstockt B, Machiels K, et al. Oncostatin M Is a Biomarker of Diagnosis, Worse Disease Prognosis, and Therapeutic Nonresponse in Inflammatory Bowel Disease. *Inflamm Bowel Dis* 2021;27(10):1564-1575. doi: 10.1093/ibd/izab032.

51. West NR, Hegazy AN, Owens BMJ, et al. Oncostatin M drives intestinal inflammation and predicts response to tumor necrosis factor-neutralizing therapy in patients with inflammatory bowel disease. *Nat Med* 2017;23(5):579-589. doi: 10.1038/nm.4307.

52. Place SP. Single-point mutation in a conserved TPR domain of Hip disrupts enhancement of glucocorticoid receptor signaling. *Cell Stress Chaperones* 2011;16(4):469-74. doi: 10.1007/s12192-010-0254-2.

53. Bai R, Shi Z, Zhang JW, et al. ST13, a proliferation regulator, inhibits growth and migration of colorectal cancer cell lines. *J Zhejiang Univ Sci B* 2012;13(11):884-893. doi: 10.1631/jzus.B1200037.

54. Wang LB, Zheng S, Zhang SZ, et al. Expression of ST13 in colorectal cancer and adjacent normal tissues. *World J Gastroenterol* 2005;11(3):336-9. doi: 10.3748/wjg.v11.i3.336.
