# Supplementary material for: Mucosal host-microbe interactions associate with clinical phenotypes in inflammatory bowel disease
Source: Nat Commun. 2024 Feb 17;15:1470. doi: 10.1038/s41467-024-45855-2 (PMC10874382; doi:10.1038/s41467-024-45855-2)
Supplement: Supplementary file 3 — Description of Additional Supplementary Files [file 41467_2024_45855_MOESM3_ESM.docx]

**Description of Additional Supplementary Files**

**File Name:** Supplementary Data 1.

**Description**: Nucleotide sequences of primers used for library construction for bacterial 16S rRNA gene (Illumina) sequencing.

**File Name**: Supplementary Data 2.

**Description**: Differential gene expression analyses between non-inflamed and inflamed biopsies from ileal CD (group 1), colonic CD (group 2) and UC (group 3).

**File Name**: Supplementary Data 3.

**Description**: Differential gene expression analysis between inflamed biopsies from patients with CD (reference) and patients with UC.

**File Name**: Supplementary Data 4.

**Description**: Differential expression analysis of deconvoluted cell types between inflamed colonic biopsies of patients with CD (reference) and patients with UC.

**File Name**: Supplementary Data 5.

**Description**: Relative abundances of mucosal bacterial groups in different groups (CD, UC and non-IBD controls) and biopsy locations (ileum or colon).

**File Name**: Supplementary Data 6.

**Description**: Comparison of relative abundances of bacterial groups between non-IBD controls and ileal CD (group 1), colonic CD (group 2) and UC (group 3).

**File Name**: Supplementary Data 7.

**Description**: Hierarchical analysis performed using an end-to-end statistical algorithm (HAllA) demonstrating the main associations between mucosal bacterial groups and clinical phenotypes.

**File Name**: Supplementary Data 8.

**Description**: Classification metrics of the predictive model performance (test dataset).

**File Name**: Supplementary Data 9.

**Description**: Genes and bacteria contained in component pairs from sparse-CCA analysis within inflamed tissues (adjusted *P*<0.05).

**File Name**: Supplementary Data 10.

**Description**: Pathway annotation of genes involved in component pairs from sparse-CCA analysis within inflamed tissues (adjusted *P*<0.05).

**File Name**: Supplementary Data 11.

**Description**: Genes and bacteria contained in component pairs from sparse-CCA analysis within non-inflamed tissues (adjusted *P*<0.05).

**File Name**: Supplementary Data 12.

**Description**: Pathway annotation of genes involved in component pairs from sparse-CCA analysis within non-inflamed tissues (adjusted *P*<0.05).

**File Name**: Supplementary Data 13.

**Description**: Individual pairwise gene-bacteria associations. (adjusted *P*<0.05).

**File Name**: Supplementary Data 14.

**Description**: Genes and bacteria associated with fibrostenotic CD/Montreal B2 (reference: Montreal B1).

**File Name**: Supplementary Data 15.

**Description**: Microbiota-associated gene clusters in patients with non-stricturing, non-penetrating disease (Montreal B1) and fibrostenotic CD (Montreal B2) including microbiota-associated pathway annotation and cluster comparisons.

**File Name**: Supplementary Data 16.

**Description**: Genes and bacteria associated with TNF-α-antagonists use (reference: non-users).

**File Name**: Supplementary Data 17.

**Description**: Microbiota-associated gene clusters in patients not using and using TNF-α-antagonists including microbiota-associated pathway annotation and cluster comparisons.

**File Name**: Supplementary Data 18.

**Description**: Individual pairwise gene-bacteria associations and their interaction with the degree of mucosal dysbiosis (Lloyd-Price et al., Nature 2019).

**File Name**: Supplementary Data 19.

**Description**: Mucosal microbiota and other phenotypic factors explaining variation in mucosal cell type enrichment in patients with IBD.
