# Supplementary material for: Mucosal host-microbe interactions associate with clinical phenotypes in inflammatory bowel disease
Source: Nat Commun. 2024 Feb 17;15:1470. doi: 10.1038/s41467-024-45855-2 (PMC10874382; doi:10.1038/s41467-024-45855-2)
Supplement: Supplementary file 5 — Reporting Summary [file 41467_2024_45855_MOESM5_ESM.pdf]

nature portfolio

Corresponding author(s): R.K. Weersma

Last updated by author(s): Dec 21, 2023

Reporting Summary

Nature Portfolio wishes to improve the reproducibility of the work that we publish. This form provides structure for consistency and transparency in reporting. For further information on Nature Portfolio policies, see our Editorial Policies and the Editorial Policy Checklist.

Statistics

For all statistical analyses, confirm that the following items are present in the figure legend, table legend, main text, or Methods section.

n/a	Confirmed	
☐	☒	The exact sample size (*n*) for each experimental group/condition, given as a discrete number and unit of measurement
☐	☒	A statement on whether measurements were taken from distinct samples or whether the same sample was measured repeatedly
☐	☒	The statistical test(s) used AND whether they are one- or two-sided<br>*Only common tests should be described solely by name; describe more complex techniques in the Methods section.*
☐	☒	A description of all covariates tested
☐	☒	A description of any assumptions or corrections, such as tests of normality and adjustment for multiple comparisons
☐	☒	A full description of the statistical parameters including central tendency (e.g. means) or other basic estimates (e.g. regression coefficient) AND variation (e.g. standard deviation) or associated estimates of uncertainty (e.g. confidence intervals)
☐	☒	For null hypothesis testing, the test statistic (e.g. *F*, *t*, *r*) with confidence intervals, effect sizes, degrees of freedom and *P* value noted<br>*Give P values as exact values whenever suitable.*
☒	☐	For Bayesian analysis, information on the choice of priors and Markov chain Monte Carlo settings
☒	☐	For hierarchical and complex designs, identification of the appropriate level for tests and full reporting of outcomes
☐	☒	Estimates of effect sizes (e.g. Cohen's *d*, Pearson's *r*), indicating how they were calculated

Our web collection on statistics for biologists contains articles on many of the points above.

Software and code

Policy information about availability of computer code

Data collection	Data from questionnaires, clinical visits and laboratory measurements were entered, checked and stored in Utopia Data Management System V.1.13.6, Research Data Support, UMCG. Data entry was performed by the trained investigators. The trained investigator who performed assessments at the study visit of a participant is responsible for data entry of that participant. All data are later checked again by the trained investigators and are subsequently stored anonymously in a secured electronic environment. The following software and packages were used: (1) Mucosal RNA-sequencing data: FastQC at default parameters (v.0.11.7), Cutadapt (v.1.1), Sickle (v.1.200), HISAT (v.0.1.6), SAMtools (v.0.1.19), flagstat and Picard tools (v.2.9.0), HTSeq (v.0.9.1), Ensemble (v.75). (2) Mucosal 16S sequencing data: Trimmomatic (v.0.33), R package DADA2 (v.1.03), silva (v.1.32). (3) Statistical analyses: R packages vegan (v.2.5-6), Compositions (v.2.02), Xcell (v.1.1.0), glmnet (v.4.1.4), microbiome (v.4.2), limma (v.4.2), ggplot2 (v.3.3.6), RColorBrewer (v.1.1.3), xgboost (v.1.6.0.1), NetCoMi (v.1.1.0).
Data analysis	Analysis was performed using R version 3.6.3. All open source software that was used is mentioned in the Methods section.

For manuscripts utilizing custom algorithms or software that are central to the research but not yet described in published literature, software must be made available to editors and reviewers. We strongly encourage code deposition in a community repository (e.g. GitHub). See the Nature Portfolio guidelines for submitting code & software for further information.

Data

Policy information about availability of data

All manuscripts must include a data availability statement. This statement should provide the following information, where applicable:

- Accession codes, unique identifiers, or web links for publicly available datasets
- A description of any restrictions on data availability
- For clinical datasets or third party data, please ensure that the statement adheres to our policy

All the analysis code is available at: https://github.com/GRONINGEN-MICROBIOME-CENTRE/Groningen-Microbiome/tree/master/Projects/IBD_biopsy_project and available via DOI: 10.5281/zenodo.10416879. Raw sequencing data and corresponding metadata that support the findings of this study are available at EGA with study ID EGAS00001002702. The participant metadata are not publicly available as they contain information that could compromise research participant privacy/consent. The authors declare that all other data supporting the findings of this study are available within this paper and its supplementary information files (including Source Data files).

Research involving human participants, their data, or biological material

Policy information about studies with human participants or human data. See also policy information about sex, gender (identity/presentation), and sexual orientation and race, ethnicity and racism.

Reporting on sex and gender	Biological sex is considered in this manuscript. All analyses were consistently adjusted for biological sex, and stratified where appropriate.
Reporting on race, ethnicity, or other socially relevant groupings	No specific reporting on race, ethnicity or other socially relevant groupings was made for this manuscript.
Population characteristics	Patients with an established diagnosis of IBD were included at the outpatient clinic of the University Medical Center Groningen (UMCG) based on their participation in the 1000IBD biobank, for which detailed phenotypic information and molecular profiles have been generated. Patients included in this study were at least 18 years old and were enrolled from 2003–2019. Diagnosis of IBD was based upon clinical, laboratory, endoscopic and histopathological criteria, of which the latter criteria also was used to determine the inflammatory status of collected biopsies. Detailed phenotypic data at time of sampling were collected for all patients.
Recruitment	Recruitment took place in the UMCG in the Netherlands. Patients were included based on their participation in the 1000IBD project (Imhann et al. BMC Gastroenterol 2019;19(1):5). Informed consent forms were available for all participants and all participants were 18 years or older at the time of sample collection.
Ethics oversight	The study protocol has been approved by the Institutional Review Board (METc 2008/338 and 2016/424, METc UMCG). Furthermore, it adheres to the UMCG Biobank Regulation, and is in accordance with the WMA Declaration of Helsinki and the Declaration of Istanbul. Study procedures were only carried out after a written informed consent was obtained.

Note that full information on the approval of the study protocol must also be provided in the manuscript.

Field-specific reporting

Please select the one below that is the best fit for your research. If you are not sure, read the appropriate sections before making your selection.

[x] Life sciences [ ] Behavioural & social sciences [ ] Ecological, evolutionary & environmental sciences

For a reference copy of the document with all sections, see nature.com/documents/nr-reporting-summary-flat.pdf

Life sciences study design

All studies must disclose on these points even when the disclosure is negative.

Sample size	Sample size was determined upon availability of sample numbers.
Data exclusions	Samples for 16S microbial characterization were further excluded by criteria total mapped reads < 2,000 through rarefaction.
Replication	Results were replicated in a publicly available dataset from Lloyd-Price et al. (Nature, 2019) and novel findings were compared to findings from experimental and small-sized human cohort studies were possible.
Randomization	N/A
Blinding	N/A

Reporting for specific materials, systems and methods

We require information from authors about some types of materials, experimental systems and methods used in many studies. Here, indicate whether each material, system or method listed is relevant to your study. If you are not sure if a list item applies to your research, read the appropriate section before selecting a response.

Materials & experimental systems

n/a	Involved in the study
☒	☐ Antibodies
☒	☐ Eukaryotic cell lines
☒	☐ Palaeontology and archaeology
☒	☐ Animals and other organisms
☐	☒ Clinical data
☒	☐ Dual use research of concern
☒	☐ Plants

Methods

n/a	Involved in the study
☒	☐ ChIP-seq
☒	☐ Flow cytometry
☒	☐ MRI-based neuroimaging

Clinical data

Policy information about clinical studies

All manuscripts should comply with the ICMJEguidelines for publication of clinical research and a completedCONSORT checklist must be included with all submissions.

Clinical trial registration	N/A
Study protocol	This study was not a clinical trial.
Data collection	All data was collected in the UMCG between 2003-2019.
Outcomes	Mucosal gut microbiota, mucosal gene expression profiles, patient outcomes (e.g. disease phenotypes, medication use).

Plants

Seed stocks	N/A
Novel plant genotypes	N/A
Authentication	N/A
